# Expression of the *Troponin C at 41C* Gene in Adult Drosophila Tubular Muscles Depends upon Both Positive and Negative Regulatory Inputs

**DOI:** 10.1371/journal.pone.0144615

**Published:** 2015-12-07

**Authors:** Maria B. Chechenova, Sara Maes, Richard M. Cripps

**Affiliations:** Department of Biology, University of New Mexico, Albuquerque, NM, 87131, United States of America; University of Valencia, SPAIN

## Abstract

Most animals express multiple isoforms of structural muscle proteins to produce tissues with different physiological properties. In Drosophila, the adult muscles include tubular-type muscles and the fibrillar indirect flight muscles. Regulatory processes specifying tubular muscle fate remain incompletely understood, therefore we chose to analyze the transcriptional regulation of *TpnC41C*, a Troponin C gene expressed in the tubular jump muscles, but not in the fibrillar flight muscles. We identified a 300-bp promoter fragment of *TpnC41C* sufficient for the fiber-specific reporter expression. Through an analysis of this regulatory element, we identified two sites necessary for the activation of the enhancer. Mutations in each of these sites resulted in 70% reduction of enhancer activity. One site was characterized as a binding site for Myocyte Enhancer Factor-2. In addition, we identified a repressive element that prevents activation of the enhancer in other muscle fiber types. Mutation of this site increased jump muscle-specific expression of the reporter, but more importantly reporter expression expanded into the indirect flight muscles. Our findings demonstrate that expression of the *TpnC41C* gene in jump muscles requires integration of multiple positive and negative transcriptional inputs. Identification of the transcriptional regulators binding the *cis*-elements that we identified will reveal the regulatory pathways controlling muscle fiber differentiation.

## Introduction

Skeletal muscle performance in higher animals is dictated in large part by the composition of their muscle fibers. A preponderance of fast fibers is found in muscles with high performance and rapid fatigue, and slow fibers predominate in muscles adapted for endurance (reviewed in [1]). These differences in fiber performance arise from differential gene expression between muscle cell types, and in particular arise from employing fiber-specific isoforms of structural muscle proteins. There is strong evidence that muscle protein isoforms are “tuned” for the physiological parameters of the fibers in which they are expressed. For example, while mutation of the *MHC-IId* Myosin heavy-chain gene in mice is partially compensated for by sustained expression of *MHC-IIa*, the mutant animals nevertheless show severe muscular defects, including growth inhibition and abnormal muscle contraction characteristics [2,3]. In addition, experimental substitution of a Drosophila flight muscle myosin isoform for an embryonic isoform results in normal myofibril assembly, but the adult flies showed muscle degeneration and an inability to fly [4].

While research over the past 30 years has identified factors required for skeletal muscle specification and development, there is still much to learn concerning how individual fiber types are specified. Clearly, the differential presence of muscle protein isoforms must arise from differential gene expression between fiber types. In zebrafish, fast and slow fiber types are physically separated from one another, making this a suitable model system for elucidating mechanisms of fiber specification. At the molecular level, the transcription factor PRDM1a represses the fast fiber phenotype by binding to the promoters of fast fiber-specific genes, and promotes slow fiber fate by suppressing *Sox6*, which otherwise represses some slow fiber genes [5]. For fast fibers, Six1a promotes the fast fiber phenotype, potentially in collaboration with myogenic bHLH factors [6]. In addition, the Pbx1 homeodomain factor also promotes fast muscle fate [7], in many cases by counteracting PRDM1a function [8]. These studies provide an important framework for understanding muscle fiber specification, and it will be important to determine if this is a broadly used pathway.

Recent research in Drosophila has demonstrated that mechanisms directing myoblasts to different fiber types indeed have evolutionarily-ancient components. The adult Drosophila thorax contains two distinct fiber types: the fibrillar indirect flight muscles that power flight, and the tubular muscles including the large tergal depressor of the trochanter (TDT), or jump muscle. These muscles differ significantly in their physiological characteristics [9], and in their patterns of structural gene expression [10,11]. Drosophila orthologs of Pbx1 and Meis1, named Extradenticle (Exd) and Homothorax (Hth), respectively, along with Spalt-major (Salm) lie at the top of a transcriptional cascade to specify flight muscle identity. Loss of function of any of these factors result in a loss of flight muscle characteristics and a transformation of the flight muscles into tubular muscle. In addition, expression of either *exd/hth* or *salm* in tubular muscles can specify flight muscle fate [10,12]. These studies therefore identify a conserved transcriptional pathway for flight muscle fate, that parallels a mechanism for fast fiber fate specification in vertebrates.

By contrast, the regulatory pathways that promote TDT fate have yet to be defined. *Myocyte enhancer factor-2 (Mef2)* is essential for formation of all adult muscles [13, 14], however no mutations or knockdowns have been identified that specifically affect the tubular class of muscle. Analyses of structural protein genes expressed in adult muscles have identified regulatory elements for a small number of genes, and some of the identified enhancers are active in the jump muscle [13, 15, 16]. Canonical MEF2 sites are present in these enhancers, although a direct role for MEF2 in regulating them has yet to be definitively shown. Overall, we still have an incomplete understanding of how tubular muscle-specific gene expression is achieved.

To address mechanisms of tubular muscle gene expression, we sought to identify and characterize the enhancer for a gene expressed exclusively in the tubular muscle, the Drosophila *Troponin C at 41C* (*TpnC41C*) gene. *TpnC41C* is one member of a family of five TpnC genes encoded by the Drosophila genome. *TpnC41C* is expressed exclusively in the tubular muscles of the adult head and thorax, and is the predominant TpnC expressed in that tissue [17,18]. By determining how the tubular muscle-specific expression of this gene is achieved, we aim to gain insight into the mechanisms specifying tubular muscle fate.

In this paper, we demonstrate that expression of *TpnC41C* in the TDT is regulated by a 300-bp promoter element that interacts with at least three different regulatory proteins. Two of these proteins, including MEF2, promote increased expression of *TpnC41C* in the jump muscles; a third factor binds to the enhancer to repress *TpnC41C* enhancer activity in the flight muscles. We therefore propose a mechanism for expression of this gene that requires the integration of both positive and negatively-acting factors to achieve proper tissue-specific expression.

## Materials and Methods

### DNA methods

Drosophila genomic DNA was isolated according to Huang et al [19] with minor modifications. A 614-bp fragment of the *TpnC41C* promoter corresponding to genomic sequence 2R:50700048–5070661 was amplified using the primers 5’-AGGGTGTATAAGCTTAGG-3’ and 5’-GCTAAATAAACAATTGAAGAC-3’ and cloned into the pGEM-T Easy vector (Promega Corp). Next, the fragment was subcloned upstream of the cytoplasmic *lacZ* reporter gene of pCHAB [20]. Note that this vector also contains a minimal heat shock promoter that provides basal promoter activity whether or not a canonical promoter is cloned into the vector. A similar strategy was applied to create plasmids with 5’- and 3’- deletions of the promoter region. To achieve this, we combined the primers 5’-CATCAAAATTCTTTATTTTTAT-3’ (for 3’ deletion) or 5’-GATGAATATTGTGTATCTAA-3’ (for 5’ deletion) with their corresponding forward and reverse primers described above. A central 300-bp part of the promoter was amplified using the primers 5’-CTACGGCCGGAGTATCAGAAGGGCGAG-3’ and 5’-GTACGGCCGATACGATGATAGCTCTGC-3’, and cloned into the EagI site of pBluescript to create pBS-TC41C-E. In addition, pTC41C-E was created, where this 300-bp promoter region was placed upstream of the nuclear *lacZ* reporter gene of pnLacZattB (provided by Dr. Basler, University of Zurich, Zurich, Switzerland), to analyze reporter expression in cell culture and in Drosophila transgenic animals.

For mutagenesis of the MEF2 binding site in the 614bp promoter and the site designated R1 in the 300bp TC41C-E, we used the GeneTailor Site-Directed Mutagenesis System (Invitrogen). The MEF2 mutation CTAA***GG***ATAA was inserted in the full-length promoter fragment using primers 5’-GATGAATATTGTGTATCTAA***GG***ATAAAACTCTG-3’ and 5’-TTAGATACACAATATTCATCAAAATTCTTTA-3’, and the mutated fragment was subcloned into pCHAB. Mutation of the R1 site in the promoter sequence (GATGAATATTGTGTA) was inserted into pBS-TC41C-E using the oligonucleotides 5’-TTATAAAAATAAAGAATTTT***TCGTCCGCGGTGTGC***TCTAAAAATAAAA-3’ and 5’-AAAATTCTTTATTTTTATAAACCGTACATT-3’; next, the mutated promoter was sub-cloned into the pnLacZattB vector to create pTC41C-ER1.

Mutations of the sites designated MEF2 and SM3 in TC41C-E (sequences CTAAAAATAA and TTCACAAATACCATTT, respectively) were generated by gene SOE-ing [21] using the mutagenic oligonucleotides 5’-GCAATTTTTG***CCTGTCTAGATTGCC*C**TATAATACGTTCCAA-3’ and 5’-ACGTATTATA***GGGCAATCTAGACAGG***CAAAAATTGCAATTC-3’ for SM3 site; oligonucleotides 5’-TATTGTGTAT***TCTGCAGCGG***AACTCTGAGTATTTT-3’ and 5’-AAAATACTCAGAGTT***CCGCTGCAGA***ATACACAATA-3’ for the MEF2 site; and oligonucleotides 5’-CGCGAATTCGAGTATCAGAAGGGCGAG-3’ and 5’-GTACGGCCGATACGATGATAGCTCTGC-3’ as common flanking primers for both mutations. Mutated enhancer PCR fragments were inserted into the EcoRI-EagI sites of pnLacZattB to create pTC41C-EMEF2, pTC41C-ESM3 and pTC41C-EMef2/SM3 plasmids. Construction of the MEF2 expression plasmid pPac-Mef2 was published elsewhere [22].

### Drosophila methods

All fly stocks were maintained at 25°C, and gene and chromosome symbols are as described at FlyBase.org. *y w* and *w* ^*1118*^ flies were used as controls. Drosophila transgenic lines expressing cytoplasmic *lacZ* controlled by the *TpnC41C* full-length promoter and its deletion variants were generated by transposon-mediated transgenesis [23]. Transgenic lines expressing nuclear *lacZ* controlled by the 300-bp enhancer and its mutant derivatives were generated by site-dependent phi-integrase mediated recombination of pTC41C-E, pTC41C-EMef2, pTC41C-ESM3, pTC41C-EMef2/SM3 and pTC41C-ER1 [24].

### Cell culture

Drosophila S2 cells were cultivated in flasks with Schneider’s Drosophila Medium (Invitrogen) supplemented with 10% FBS (Atlanta Biologicals), at 24°C to a density of 2-4X10^6^ cells/ml. For co-expression of Drosophila MEF2 protein and β-galactosidase controlled by full-length *TpnC41C-lacZ* and its deletion variants, cells were plated into 24-well plates at 5X10^5^ cells per well and incubated with DNA and Cellfectin II Reagent (Invitrogen) in serum-free Schneider’s Drosophila Medium for 18 h at 24°C. Total plasmid DNA concentration per well was 0.5 mg/ml, with a transcription factor (MEF2) to reporter (*TpnC41C-lacZ*) plasmid ratio of 1:9. Next, an equal volume of serum-containing culture medium was added, and cells were allowed to grow for an additional 24 h at 24°C. After this time, growing media was removed from the wells, cells were lysed, and lysates were used in β-galactosidase assays.

### β-galactosidase assay

We analyzed the amount of expressed β-galactosidase reporter protein by measuring its activity in a colorimetric reaction using the All-in-One β-Galactosidase Assay reagent (Pierce). For β-galactosidase activity in cell culture, cells were lysed in 100 μl of M-PER buffer (Pierce) and incubated for 20 min at room temperature with shaking. Next, 20 μl aliquots of lysates were mixed with equal amount of β-galactosidase reagent, and reporter activity was measured at 405 nm in a Multiskan FC plate reader (Thermo Scientific). The resulting activity was normalized to the total protein concentration in the lysates, measured with the Bio-Rad Protein Assay Reagent.

For β-galactosidase activity in transgenic flies, ten dissected TDT muscles were homogenized in 100 μl of PBTx. All insoluble debris were sedimented by centrifugation, and 30 μl aliquots of the supernatant were mixed with 10 μl of the β-galactosidase reagent. The reactions were analyzed immediately by taking ten measurements every 2 minutes in the Multiskan FC plate reader at 405 nm. Resulting activity was calculated as a change in the light absorbance per second. The results were expressed as a percent of β-galactosidase activity relative to that observed in transgenic control muscles.

### Electrophoretic mobility shift assay and transcription factor pull-down experiment

DNA binding assays were carried out as described by Cripps et al [25]. For MEF2-binding reactions, MEF2 protein was synthesized in a Coupled Rabbit Reticulocyte Lysate Transcription-Translation System (Promega) using pcDNA-Mef2 [26] as a DNA template and T3 RNA Polymerase. The *TpnC41C* putative MEF2 binding site, plus ten nucleotides on each side, was used as a binding DNA probe. Double-stranded oligonucleotides corresponding to this region included 5’-GG overhangs necessary for incorporation of radioactive ^32^P-dCTP in a Klenow Exo^-^ enzymatic reaction (New England Biolabs). For the competitor-containing reaction we used 100X excess of unlabeled DNA probe. The mutated competitor had a two-nucleotide substitution in the MEF2 binding site. Binding reactions were carried out in binding buffer (25mM HEPES pH 7.6, 100 mM NaCl, 15% Glycerol, 0.1% NP40, 0.5 mM PMSF; [27]) supplemented with Complete EDTA-free protease inhibitor cocktail (Roche) and 0.1 μg/μl poly-dIdC, and containing 1μl of either unprogrammed or programmed lysate. First, all components besides the labeled probe were mixed and incubated on ice for 20 min. Then, the probe was added and reactions were incubated at room temperature for additional 20 min. Reaction components were resolved in 5% non-denaturing polyacrylamide gels, and visualized by autoradiography of the dried gel.

To explore the *TpnC41C* promoter for new protein binding sites, we designed 50-bp overlapping probes that completely covered its sequence. Nuclear protein extracts from *w* ^*1118*^ pupae were prepared for binding reactions according to Kawasaki et al [28], and 2μg of protein was used in each reaction. Specific protein binding to the 30-bp sequence 5’-ATTTTGATGAATATTGTGTATCTAAAAATA-3’ was confirmed in an additional assay. Mutated probes *m1-m6* were designed to define the 15-bp binding site.

Pull down experiments were performed using Dynabeads MyOne Streptavidin T1 magnetic beads (Invitrogen) and double-stranded 170-bp biotinylated *TpnC41C* promoter region (bait), containing either the native or the mutated MEF2 binding site. Baits were amplified using the biotinylated forward primer 5’-Biotin-ATTATAGCGTTCGCTCTTG-3’ and the non-biotinylated reverse primer 5’-AATTTTATCCACAAGACAG-3’, and plasmids pTC41C-E and pTC41C-EMef2 as templates. For the binding reactions, we scaled up the pupal nuclear extract preparation to obtain ~ 5mg of total protein per sample. The nuclear extract buffer was further exchanged with binding buffer described above using Amicon Ultra Centrifugal Filters (Millipore), and incubated with 250 μl of salmon sperm DNA (10mg/ml) at 4°C for 30 min to block non-specific DNA-protein binding. Next, the nuclear extract was incubated with magnetic beads carrying baits at 4°C for 2 h. After incubation, beads were washed twice with binding buffer containing protease inhibitors, and twice with 20mM Tris-HCl, pH 8.0, 2mM CaCl2, and bound proteins were digested with trypsin directly on the beads as described in Belozerov et al [29]. The products of the enzymatic digest were submitted to the Arizona Proteomics Consortium (University of Arizona) for liquid chromatography-tandem mass spectrometry.

### Cryosectioning, immunofluorescence and microscopy

Cryosectioning of adult flies, immunostaining and histochemical staining for β-galactosidase were performed as described before [30]. Antibody to β-galactosidase was obtained from Promega. We used Alexa Fluor IgG conjugates (Molecular Probes) as secondary antibodies in all immunofluorescent stains. Alexa Fluor-conjugated Phalloidin (Molecular Probes) was used to visualize F-actin in muscle cells. For direct comparison of X-Gal stains between control transgenic lines and mutant transgenic lines, sections were treated and incubated with X-Gal solution for identical periods of time. Images for direct comparisons between control and experimental samples were collected under identical conditions, and image processing was identical for all matched images.

## Results

### The *TpnC41C* promoter is conserved in the Melanogaster subgroup and contains sequences sufficient for tissue-specific expression

**Fig 1:**
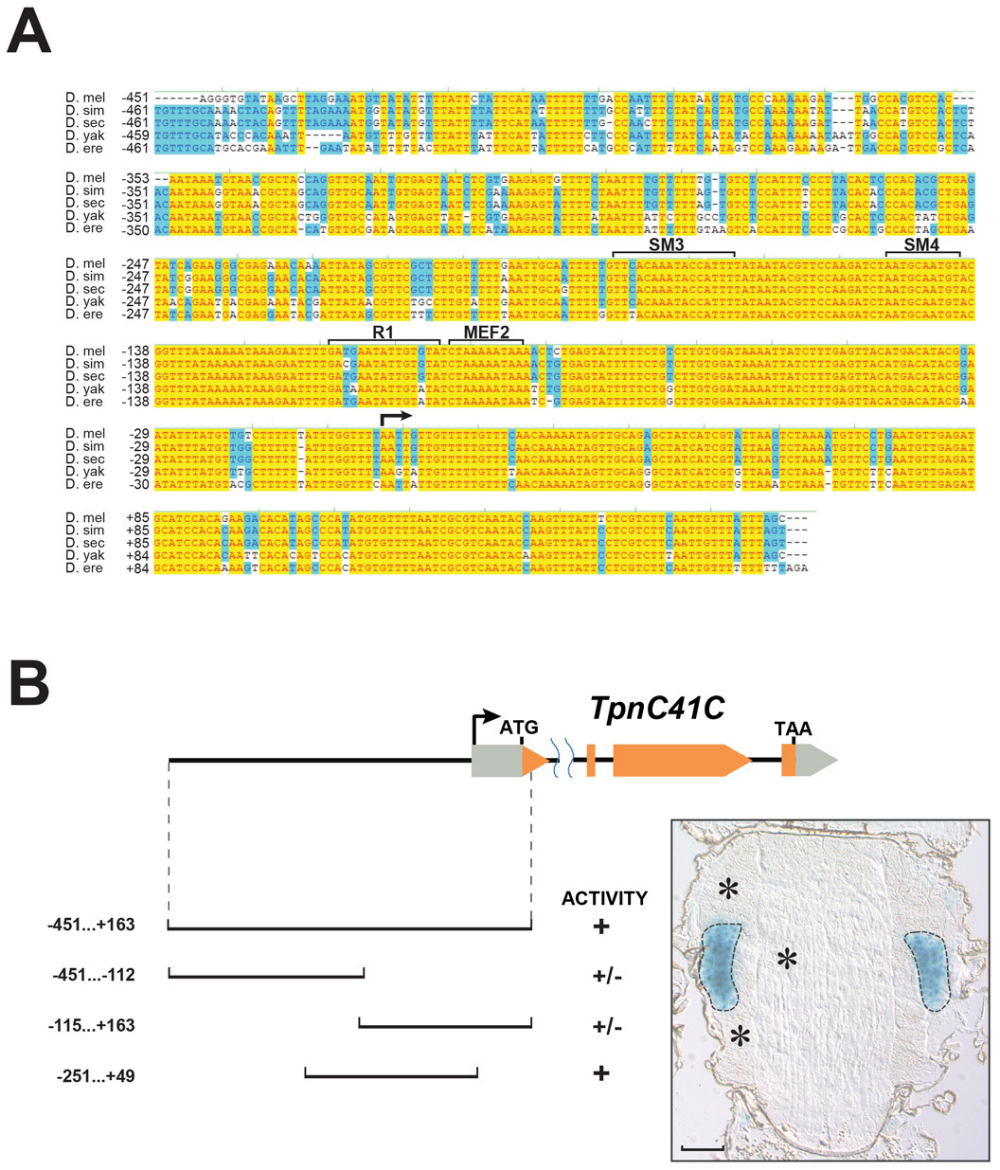
The *TpnC41C* promoter is conserved in the Sophophora subgenus and specifically drives reporter expression in the TDT. (A) The high conservation of the upstream region of the *TpnC41C* gene is revealed by sequence alignment across five Drosophila species: *D*. *melanogaster (D*. *mel)*, *D*.*simulans (D*. *sim)*, *D*.*sechellia (D*. *sec)*, *D*. *yakuba (D*. *yak)* and *D*.*erecta (D*. *ere)*. Fully conserved nucleotides are highlighted in yellow, and strongly conserved nucleotides are highlighted in blue. Arrow shows the location of the transcription start site, and numbers indicate nucleotide positions relative to the transcription start at +1. Brackets show localization of the conserved binding sites which are discussed later in the paper. (B) Schematic of the *D*. *melanogaster TpnC41C* gene, showing exon composition. Protein coding regions are shown in orange, untranslated regions shown in grey. Below are diagrams of the promoter regions tested for activity in the TDT using a *lacZ* reporter. Promoter activity was evaluated by X-Gal staining on cryosections and was classified as “strong” (+), when the blue staining developed in 5 min; or “weak” (+/-) when it developed in more than 1 h. Right panel shows a representative stained section of the Drosophila thorax expressing the *nlacZ* reporter controlled by a 300-bp promoter region TC41C-E (spanning the region from -251 to +49). TDT muscles are outlined with a dashed line, asterisks indicate indirect flight muscles. Bar, 100μm.

To identify the genomic elements controlling expression of *TpnC41C*, we searched the DNA sequence immediately upstream of this gene for regions that are conserved across multiple Drosophila species. We found that sequence in the immediate proximity to the transcription start site has 76% nucleotide identity across five species of the Melanogaster subgroup to which *D*. *melanogaster* belongs (Fig 1A). To determine if this region can account for the tissue-specific expression of *TpnC41C*, we cloned a ~600-bp fragment from the identified homologous region and fused it to *hsp-lacZ*, that encodes β-galactosidase. We monitored the expression of this reporter construct in transgenic animals, where we observed it to be sufficient to direct strong and specific expression of β-galactosidase in the TDT muscles (Fig 1B), which indicated that the cloned region contained all the regulatory sequences required for fiber-specific expression of a muscle structural gene. To gain further insight into how this region was regulated, we generated three sub-fragments, and tested their ability to activate an *hsp-lacZ* reporter in transgenic animals. We found that whereas the 5’ and 3’ halves had only modest activity in the TDT, a central fragment spanning from -251 to +49, that we termed TC41C-E, retained strong TDT-specific activity (Fig 1B, right panel). These results defined more precisely the location of *TpnC41C* regulatory elements.

### The *TpnC41C* promoter contains a functional binding site for MEF2

**Fig 2:**
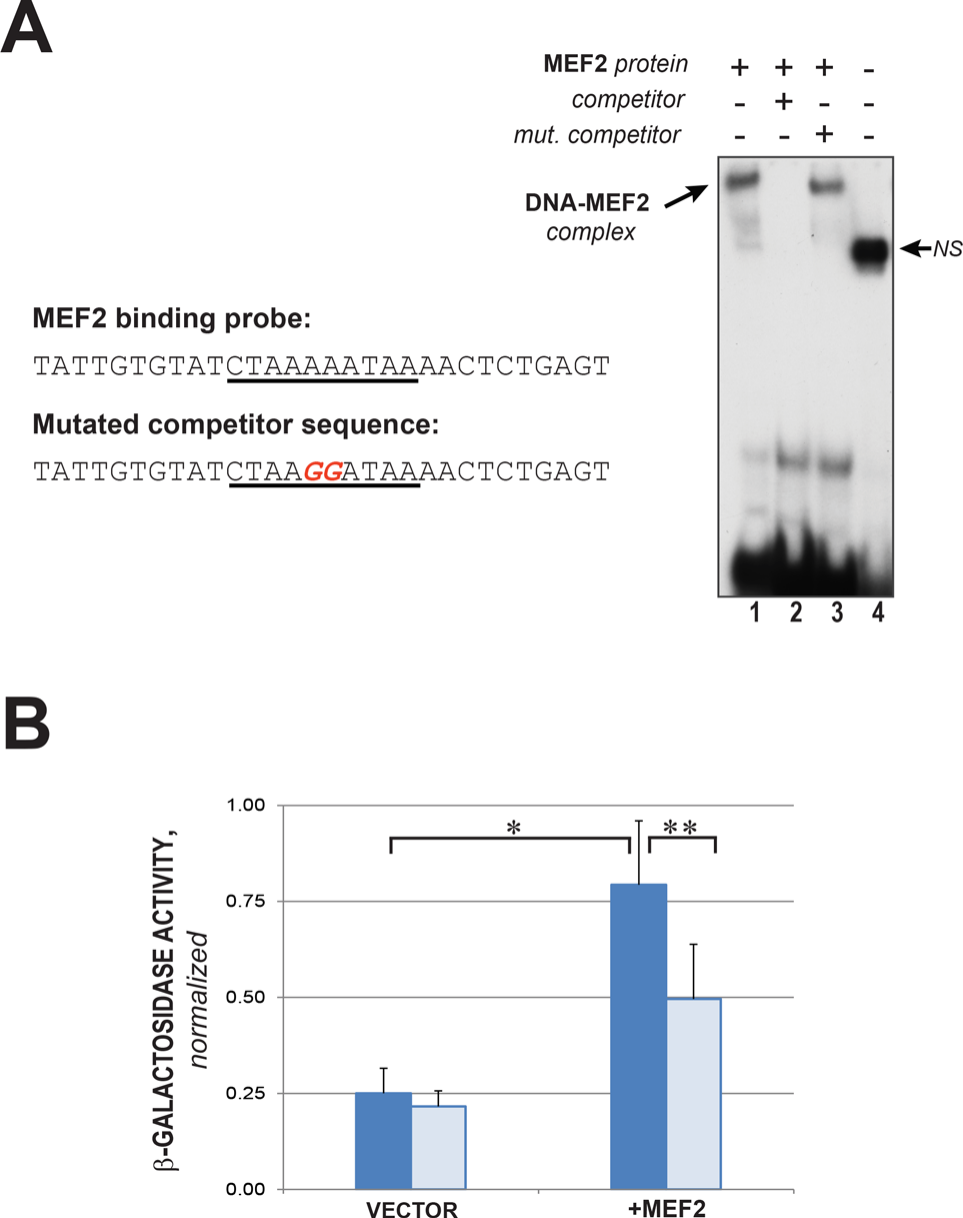
MEF2 binds to and activates the *TpnC41C* promoter. (A) MEF2 binding to the *TpnC41C* promoter was analyzed by electrophoretic mobility shift assay. On the left: the sequence of the 30-bp probe, containing a putative MEF2 site (underlined) and its mutated version (mutated nucleotides shown in red). The double-stranded, radioactively-labeled wild-type probe was incubated with *in vitro* translated MEF2 protein, resulting in a shifted DNA-MEF2 complex (lane 1). The specificity of binding was tested by adding to the reaction mixture a 100-fold excess of the same unlabeled oligonucleotide (“competitor”, lane 2), or unlabeled oligonucleotide with the nucleotide substitution in the MEF2 site (“mut. competitor”, lane 3). Lysate containing no MEF2 protein only resulted in a non-specific interaction between protein in the lysate and the DNA probe (lane 4, NS). (B) Activation of the *TpnC41C* promoter in Drosophila S2 cells by MEF2. Cells were transfected with either wild type (dark blue) or mutated (light blue) *TpnC41C* promoter-*nlacZ* constructs, alongside either empty expression vector (VECTOR) or expression vector for MEF2 (+MEF2). β-galactosidase activity was analyzed in cell lysates 24 hours post-transfection, and the results are expressed as normalized β-galactosidase activity. Asterisks indicate t-test p-values; * p = 2.18x10^-5^, ** p = 0.003283.

To determine how the tissue-specific activity of the *TpnC41C* promoter was regulated, our initial goal was to identify sequences that were conserved within the promoter. In practice, this was not possible since the promoter is highly conserved across the species tested, and orthologous sequences could not be identified in more distantly-related Drosophila species. Instead, we examined the sequence for canonical binding sites for known muscle regulatory factors. We found that it contained a putative binding site for the Drosophila myogenic transcription factor, Myocyte enhancer factor-2 (MEF2)(Fig 2A), that matched the consensus MEF2 binding site identified by Andres et al [31], and closely matches MEF2 binding sites identified in a number of Drosophila studies [32–34]. This site was present and conserved in all five other Drosophila species (see Fig 1A).

To test if MEF2 can bind to this site, we performed an electrophoretic mobility shift assay (EMSA). We found that *in vitro* translated MEF2 bound to this sequence, and the binding was sensitive to mutations in the MEF2 consensus binding site and therefore sequence-specific (Fig 2A). This *in vitro* result was consistent with a role for MEF2 in regulating the promoter.

To determine if this sequence could interact with MEF2 isolated from intact animals, we used a 170-bp fragment of the *TpnC41C* promoter, containing the MEF2 site, as a bait to isolate nuclear proteins prepared from pharate adults. In parallel, we mutated the MEF2 site in the context of the 170-bp fragment and used it as a negative control for the pull-down experiment. The purified nuclear protein samples were then analyzed using LC-MS/MS analysis to identify factors interacting with the control and mutant DNA fragments. All Drosophila proteins identified in this approach are listed in S1 Table. Amongst the identified peptides, MEF2 was detected in the samples purified using wild-type DNA (seven peptide hits), but was not detected in samples using mutant DNA (0 peptide hits). This result indicated that MEF2 from intact animals could interact specifically with the *TpnC41C* promoter fragment. We note that a number of other proteins are co-purified in this approach, including a number of muscle structural proteins that are unlikely to interact with DNA. Further optimization of this approach should identify additional factors interacting specifically with the *TpnC41C* promoter, and reduce the background of non-specific proteins.

We next validated the function of the identified MEF2 site in tissue culture and *in vivo*. We co-transfected Drosophila S2 cells with a *Mef2* expression plasmid and full-length *TpnC41C-lacZ*. This resulted in activation of *lacZ* expression, evident from the increased β-galactosidase activity in cell lysates compared to control co-transfections containing an empty expression plasmid plus full-length *TpnC41C-lacZ*. A similar co-transfection experiment with *Mef2* expression plasmid and mutated *TpnC41C-lacZ*, in which the MEF2 site was altered, had a significant reduction in β-galactosidase activity compared to the control fragment plus MEF2 (Fig 2B). This result indicated that the MEF2 site that we had identified was critical to full MEF2-mediated activation of the enhancer. Interestingly, there was still some activation by MEF2 of the mutated *TpnC41C-lacZ* reporter. This result might arise from MEF2 interacting with the promoter via a non-consensus binding site and thereby partially activating reporter gene expression.

**Fig 3:**
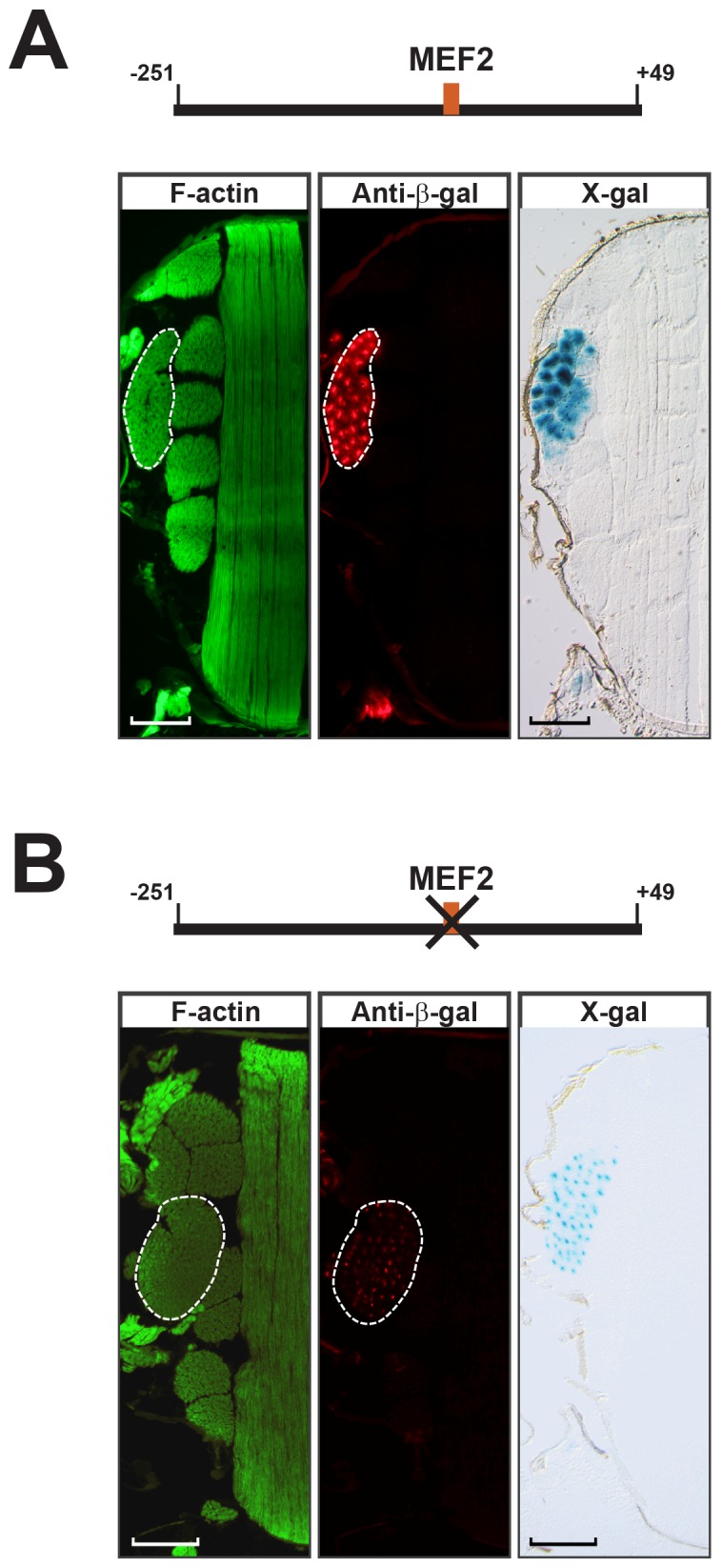
MEF2 regulates tissue-specific activity of the *TpnC41C* promoter. (A, B) Diagrams of the *TpnC41C* promoter-*nlacZ* constructs tested, with the position of the wild type (A) or mutated (B) MEF2 binding sites indicated. Below each diagram are representative cryosections of the corresponding transgenic flies, stained with: Phalloidin (green, left panels), to reveal the thoracic muscles; anti-β-galactosidase (red, center panels), to reveal nuclear β-galactosidase accumulation; and X-Gal (blue, right panels), to reveal nuclear β-galactosidase activity. The TDT is outlined. Scale bar, 100μm.

I*n vivo*, we analyzed the activities of wild-type and MEF2 site mutated TC41C-E promoters fused to a nuclear *lacZ* reporter. These reporters were inserted into independent lines at the same genomic locus, thus eliminating possible variation in expression due to positional effect. Samples were also processed at the same time under identical conditions. We observed that whereas the wild-type promoter-*nlacZ* construct was strongly active in the jump muscles (Fig 3A), mutation of the MEF2 site substantially reduced the *TpnC41C* promoter activity (Fig 3B).

Altogether, these findings established MEF2 as a direct regulator of *TpnC41C*, acting via a single binding site in the *TpnC41C* promoter and thereby playing an important role in activation of this gene in the TDT.

### The *TpnC41C* promoter contains binding sites for additional transcriptional activators

**Fig 4:**
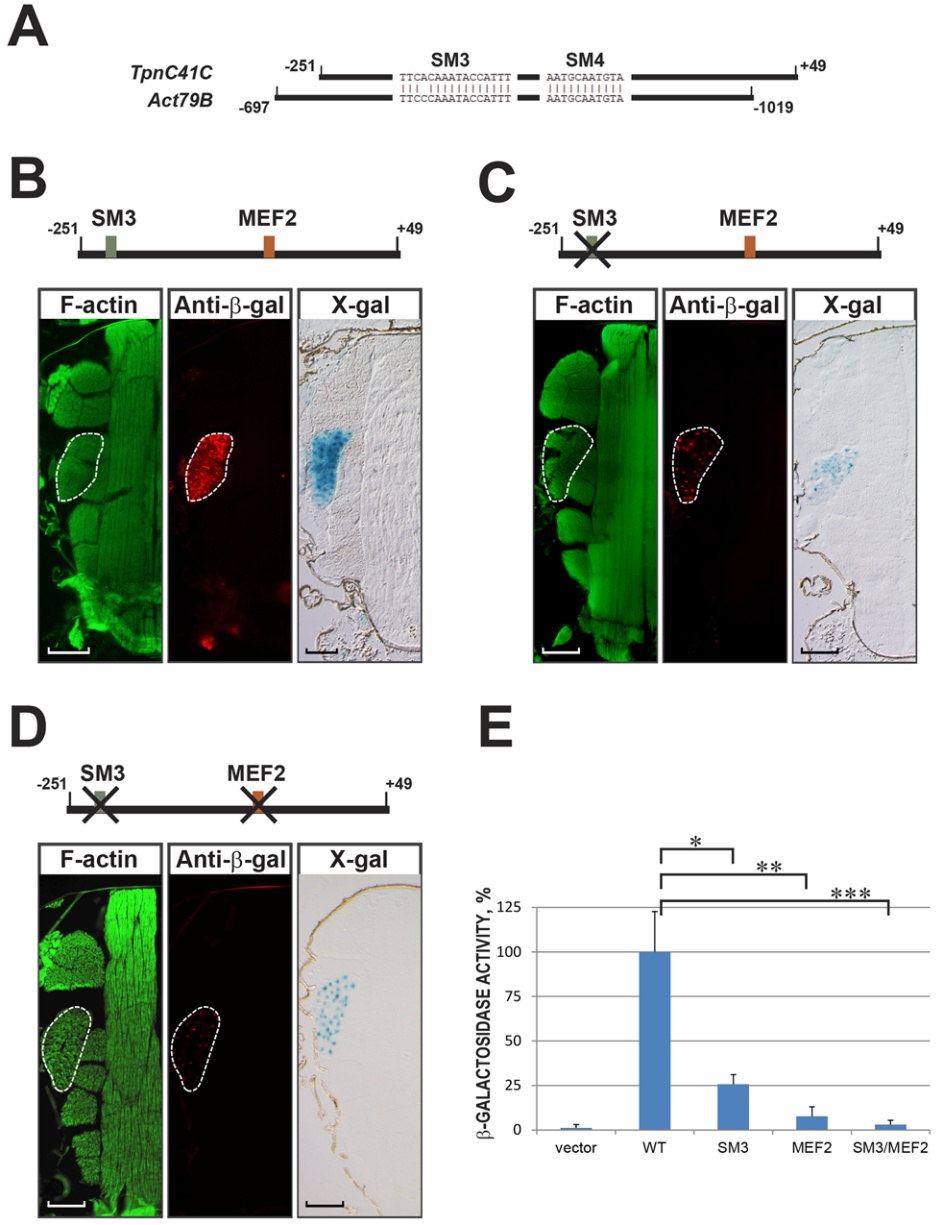
Additional positive regulatory elements exist in the *TpnC41C* promoter. (A) Diagram of the aligned *TpnC41C* and *Act79B* regulatory regions, showing the similar sequences termed SM3 and SM4. (B-D) Top: diagrams of *TpnC41C* promoter-*nlacZ* constructs bearing either wild-type (B), mutated SM3 (C) or double mutant SM3 and MEF2 sites (D). Below the schematics are representative images of cryosections of the transgenic flies stained with: Phalloidin (green, left panels), to reveal the thoracic muscles; anti-β-galactosidase (red, center panels), to reveal nuclear β-galactosidase accumulation; and X-Gal (blue, right panels), to reveal nuclear β-galactosidase activity. The TDT is outlined. Scale bar, 100μm. (E) β-galactosidase activity was measured in lysates from dissected TDT muscles expressing either the *nlacZ* reporter with no enhancer (vector) or *nlacZ* reporters controlled by wild type (WT) and mutant *TpnC41C* promoters (SM3 or SM3/MEF2 indicating the sites that were mutated). Asterisks indicate t-test p-values; * p = 2.2268x10^-12^, ** p = 4.49158x10^-18^, *** p = 5.39191x10^-19^.

Since mutation of the MEF2-binding site did not completely eliminate transcriptional activity of TC41C-E, we searched for other possible regulatory sequences in the *TpnC41C* promoter. In this approach, we searched for homologous sequences in the promoters of other TDT-specific muscle structural genes. We hypothesized that expression of genes in the same set of tissues might involve similar mechanisms of regulation, and therefore the enhancers would contain similar regulatory elements. When we compared the sequence of the *TpnC41C* promoter with that of the enhancer of the jump muscle actin gene *Act79B* [13], we identified two regions of significant similarity. The two identified regions, that we named SM3 and SM4, demonstrated 93% and 100% nucleotide identity, respectively (Fig 4A).

To determine the functional importance of these two sequences, we mutated each of them in the context of the 300-bp *TpnC41C* promoter, and assessed promoter-*lacZ* activity in transgenic animals in comparison to wild-type *TpnC41C-nlacZ* samples processed in parallel (Fig 4B). Mutation of SM3 resulted in visible reduction of β-galactosidase levels in the jump muscles of transgenic flies (Fig 4C). In contrast, mutation of SM4 in the same setting did not result in a noticeable changes in the normal reporter expression (not shown). These data indicated that SM3, like the MEF2 site, is critical for full activation of *TpnC41C* promoter activity, and suggested that SM3 represents a binding site for a transcriptional regulator that activates *TpnC41C* expression in jump muscles.

To determine if the MEF2 and SM3 sites were in combination critical for enhancer activity, we generated a double mutation affecting both the SM3 and MEF2 sites. *In vivo*, this mutant promoter-*nlacZ* reporter also showed low, but detectable, reporter gene expression (Fig 4D).

To determine more accurately the requirements of the MEF2 and SM3 sites for *TpnC41C* promoter activity, we carried out quantitative analyses of β-galactosidase activity in the lysates of transgenic flies bearing wild-type and mutant versions of TC41C-E. Mutation of SM3 resulted in a significant reduction of β-galactosidase activity, reducing it to 26% of the wild type activity level, whereas mutation of the MEF2 site and double mutation of MEF2/SM3 caused more dramatic changes, practically eliminating reporter activity (8% and 3% of wild-type reporter activity, respectively) (Fig 4E).

Our data demonstrate that although MEF2 is seemingly the most significant regulator of the *TpnC41C* promoter, driving most of its activity in the jump muscle, additional factors contribute to the positive regulation of *TpnC41C* transcription.

### The *TpnC41C* promoter contains a repressor binding site

**Fig 5:**
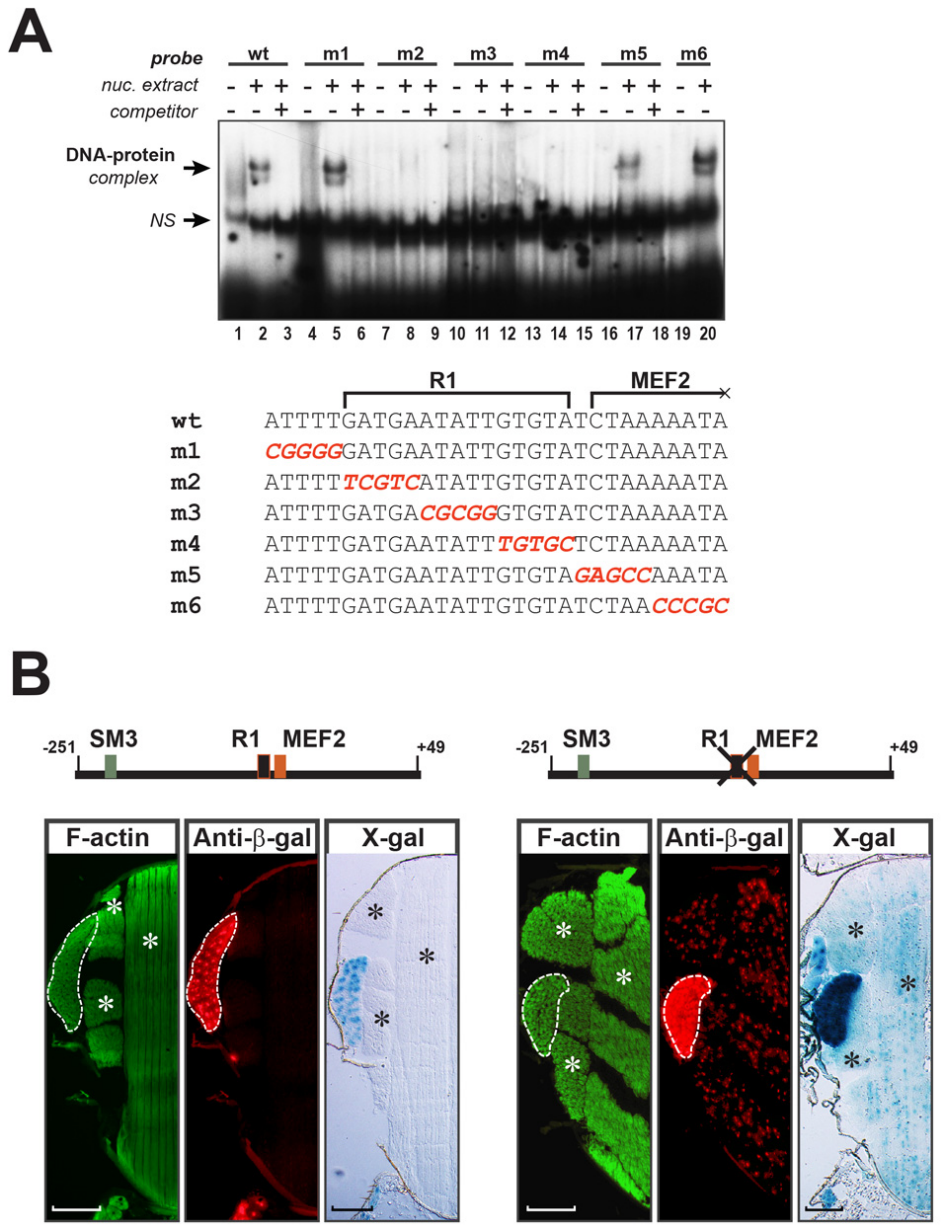
Identification of a negative regulatory element within the *TpnC41C* enhancer. (A) The upper panel shows an electrophoretic mobility shift assay resulting from combination of pupal nuclear extracts with dsDNA probes. For each probe, lanes contain radioactively-labeled probe alone (lanes 1, 4, 7, 10, 13, 16, 19), or probe plus nuclear extract (lanes 2, 5, 8, 11, 14, 17, 20), or probe plus nuclear extract plus 100-fold excess of unlabeled probe (lanes 3, 6, 9, 12, 15, 18). NS–non-specific band. Probe sequences are shown in the lower panel. Red sequences indicate mutated nucleotides. Brackets indicate boundaries of the R1 and the truncated MEF2 sites.(B) Mutation of R1 boosts promoter activity and compromises specificity of the *TpnC41C* promoter fragment. Upper diagrams show relative position of the R1 site within the promoter-*nlacZ*. Below the diagrams are representative images of cryosections of the transgenic flies stained with: Phalloidin (green, left panels), to reveal the thoracic muscles; anti-β-galactosidase (red, center panels), to reveal nuclear β-galactosidase accumulation; and X-Gal (blue, right panels), to reveal nuclear β-galactosidase activity. The TDT is outlined, and asterisks indicate the flight muscles. Scale bar, 100μm.

Since MEF2 is expressed in all adult muscles [35], our results so far did not give insight into how *TpnC41C* is expressed only in the jump muscles. We therefore sought to identify additional regulatory elements in the *TpnC41C* promoter, by performing EMSA using nuclear extracts prepared from developing pupae. By using a series of 30- and 50-bp DNA probes, collectively overlapping the entire length of the cloned *TpnC41C* promoter, we identified a 30-bp region that produced a reproducible protein-DNA complex when combined with nuclear extracts (Fig 5, lanes 1–3). Note that this region is immediately adjacent to the MEF2 binding site, and part of, although not all of, the MEF2 site is contained within the 30-bp sequence.

To identify sequences within this 30-bp element that interact with the DNA binding protein, we generated mutant versions of the probe, in which consecutive stretches of five nucleotides were mutated (see lower part of Fig 5A). We observed that mutation of either the first five nucleotides (Fig 5A lanes 4–6), or of the last ten nucleotides (Fig 5A lanes 16–20), did not affect the formation of a DNA-protein complex. By contrast, mutation of the inner 15 nucleotides prevented binding of the protein to DNA (Fig 5A lanes 7–15). This 15-bp sequence, that we termed R1, is different from the other characterized regulatory elements in the *TpnC41C* enhancer described above.

Given that this sequence binds a protein from pupal nuclear extracts, we determined the requirement for this element in *TpnC41C* enhancer activity. We mutated R1 in the context of the full-length enhancer, and fused it to *nlacZ* for analysis in transgenic animals. Control and mutant transgenic *TpnC41C-nlacZ* animals were processed for immunofluorescence and immunohistochermistry in parallel and under identical conditions. Interestingly, mutation of this sequence in the context of TC41C-E resulted in up-regulation of the reporter activity, as evident from stronger accumulation of β-galactosidase in the jump muscles compared (Fig 5B). More importantly, ectopic β-galactosidase accumulation was detected in the indirect flight muscles of the mutant *TpnC41C-nlacZ* samples. These data suggested that a transcriptional repressor, acting via R1, is involved in proper fiber-specific expression of *TpnC41C*.

Taken together, the data presented in this report indicate that fiber-specific expression of *TpnC41C* is regulated by a combination of positive and negative regulatory inputs.

## Discussion

In this paper, we have identified and characterized the mechanisms controlling transcription of the Drosophila tubular muscle structural gene, *TpnC41C*. Expression of this gene is controlled by a proximal enhancer located close to the basal promoter, and that is regulated by both positive and negative regulatory inputs. Specifically, the myogenic regulator MEF2 plus one other factor promote *TpnC41C* expression, whereas a third factor functions as a repressor of *TpnC41C* expression.

### Programming of tubular muscle fate

What do our findings tell us about the mechanisms specifying tubular muscle fate? Currently there is little information regarding genes that specifically control the formation of the tubular muscles in the Drosophila adult. *Mef2* knockdowns lack essentially all adult skeletal muscles [13, 14], and knockdown of either *spalt-major*, *exd* or *hth* result in a loss of flight muscle fate and an expansion of tubular muscle fate into the flight muscles [10,12]. However, no factors have to date been shown to be required solely for tubular muscle fate. These data raise two possible conclusions: that a genetic program exists for specifying tubular muscle fate, but this program has yet to be defined; or that tubular muscle fate is a default process, and additional factors expressed in flight muscles (such as Exd/Hth and Spalt-major) repress jump muscle fate and instead promote flight muscle fate.

Here, we demonstrate that a TDT-specific regulatory element can expand its expression into the flight muscles when a specific sequence is mutated. This finding is similar to that of Arredondo et al [36] who showed that the activity of a 1.7-kb promoter element within the *Paramyosin* gene had an expansion of enhancer activity from the TDT into the flight muscles, when a number of regulatory sites were mutated. These two studies support a model for tubular muscle enhancer architecture where the activity of a general muscle activator region is attenuated in specific muscle sub-types such as the flight muscles. Such a model of enhancer activity would support the latter model of jump muscle specification: a default developmental pathway promotes tubular muscle fate, and factors expressed in the flight muscles repress tubular muscle fate and instead promote flight muscle fate. Nevertheless, we note that this is a relatively limited analysis upon which to derive a firm conclusion.

### Role of MEF2 in controlling adult muscle gene expression

For the *TpnC41C* promoter, it is clear that one general muscle activator is the MADS domain protein MEF2, that works in collaboration with at least one additional factor. This situation differs somewhat from the *Paramyosin* internal promoter, since a putative MEF2 site was one of the sequences whose mutation resulted in enhancer activity expanded outside the jump muscle. In addition, several other adult tubular muscle enhancers appear to lack consensus MEF2 sites, including the 5’ *Paramyosin* enhancer [36] and a sub-fragment of the upstream regulatory element of the Troponin T gene, *upheld* [15]. These studies indicate that factors in addition to MEF2 must be required to promote tubular muscle gene expression, however the identity of these factors remains obscure.

The presence of a single MEF2 site in a regulatory element has been observed before in a number of instances, including for the *Tm2* gene [37] and *Actin57B* [32] among others. In these two cases, additional regulatory elements are necessary for full enhancer activity [37, 22], much like we observe here for *TpnC41C*. In other instances, multiple MEF2 sites have been identified within regulatory elements (see for example [38]). It is currently not apparent why such different enhancer architectures might exist. Our data also indicate that in tissue culture assays MEF2 can cause modest activation of *TpnC41C-lacZ* even when the MEF2 site is mutated. This may arise from MEF2 interacting with a non-canonical site, as has been previously observed for MEF2, in particular when activation of the target gene occurs through collaboration of MEF2 with co-factors [39].

A method we used to identify regulators of *TpnC41C* was to use the promoter to purify DNA binding factors from nuclear extracts. Using this approach, we identified MEF2 as a factor bound to the wild-type sequence, that was not bound to a MEF2-mutated version of the promoter. This provided strong support for our data suggesting that MEF2 was a regulator of *TpnC41C*. On the other hand, while we hoped that this approach might identify additional regulators of the *TpnC41C* promoter, relatively few additional DNA-binding factors interacted with the sequences used as bait, and a number of muscle structural proteins were also detected (see S1 Table). Therefore, while this approach has promise for identifying novel transcriptional regulatory factors, it still needs to be optimized. One possible avenue for optimization is to carry out a more quantitative analysis of protein binding to the promoter fragment, using stable isotope labeling by amino acids in cell culture (SILAC)[40]. This approach has been optimized for use in developing Drosophila [41].

### Proximity of the *TpnC41C* repressor site to the MEF2 site

While we have yet to identify the factor that suppresses *TpnC41C* promoter activity in the flight muscles, we have localized its binding site to a position close to the MEF2 site that is required for maximum gene activation. One potential mechanism for repression by this factor is that it is expressed in non-tubular muscles such as the flight muscles, and functions by sterically blocking MEF2 from interacting with its site. Among many other examples, this mechanism is similar to that employed by Sp3 in inhibiting Sp1-mediated gene activation (reviewed in [42]).

We also note that mutation of the repressor site, termed R1, causes an increase in promoter activity in the jump muscles, suggesting that the repressor may be expressed strongly in non-tubular muscles, but also at low levels in the TDT. One candidate repressor may be the zinc finger transcription factor Spalt-major, that is detected in the flight muscles and functions there to suppress jump muscle fate [10], and that also is expressed at low levels in the jump muscles [12]. However, the published DNA binding specificity for Spalt proteins [43] does not match closely the sequence of R1.

An alternative interpretation of the effects of R1 mutation is that the R1 repressor site represents a location at which promoter activity is globally repressed, and jump muscle-specific gene expression is achieved through over-riding such repression. Such an interpretation would imply that the *TpnC41C-lacZ* reporter is active in the flight muscles, albeit at very low levels, and that mutation of R1 simply enables higher levels of reporter expression in both muscle types. Although in our experiments we never observed specific expression from the wild-type *TpnC41C-lacZ* reporter in the flight muscles, a recent paper reported low levels of TpnC41C in IFMs [44]. This fact can be an argument in favor of the second model. Overall, our studies represent a detailed analysis of the sequences that control expression of a tubular muscle-specific gene in Drosophila, and uncover mechanisms for how tubular muscle fate might be specified.

## Supporting Information

S1 Table Proteins identified binding to wild-type (WT) and MEF2 site-mutated (MEF2) *TpnC41C* regulatory sequences. (XLSX)

## References

[1] Lieber RL. Skeletal muscle structure, function, and plasticity, 3rd edition Lippincott Williams & Wilkins 2010.

[2] Sartorius CA, Lu BD, Acakpo-Satchivi L, Jacobsen RP, Byrnes WC and Leinwand LA. Myosin heavy chains IIA and IId are functionally distinct in the mouse. J Cell Biol 1998; 141: 943–953. 9585413 10.1083/jcb.141.4.943 PMC2132782

[3] Allen DL, Leinwand LA. Postnatal myosin heavy chain isoform expression in normal mice and mice null for IIb or IId myosin heavy chains. Dev Biol 2001; 229: 383–395. 11150240 10.1006/dbio.2000.9974

[4] Wells L, Edwards KA, Bernstein SI. Myosin heavy chain isoforms regulate muscle function but not myofibril assembly. EMBO J. 1996; 15: 4454–4459. 8887536 PMC452174

[5] Von Hosfstren J, Elworthy S Gilchrist MJ, Smith JC, Wardle FC, Ingham PW. Prdm1- and Sox6-mediated transcriptional repression specifies muscle fiber type in the zebrafish embryo. Development 2008; 138: 4399–4404. 10.1038/embor.2008.73 PMC2424280 18535625

[6] Bessarab DA, Chong S-W, Srinivas BP, Korzh V. Six1a is required for the onset of fast muscle differentiation in zebrafish. Dev Biol 2008; 323: 216–228. 10.1016/j.ydbio.2008.08.015 18789916

[7] Maves L, Waskiewicz AJ, Paul B, Cao Y, Tyler A, Moens CB et al Pbx homeodomain proteins direct MyoD activity to promote fast-muscle differentiation. Development 2007; 134: 3371–3382. 17699609 10.1242/dev.003905

[8] Yao Z, Farr GH III, Tapscott SJ, Maves L. Pbx and Prdm1a transcription factors differentially regulate subsets of the fast skeletal muscle program in zebrafish. Biol Open 2013; 2: 546–555. 10.1242/bio.20133921 23789105 PMC3683157

[9] Peckham M, Molloy JE, Sparrow JC, White DC. Physiological properties of the dorsal longitudinal flight muscle and the tergal depressor of the trochanter muscle of *Drosophila melanogaster* . J. Muscle Res. Cell Motil. 1990; 11: 203–215. 2119393 10.1007/BF01843574

[10] Schonbauer C, Distler J, Jahrling N, Radolf M, Dodt HU, Frasch M et al Spalt mediates an evolutionarily conserved switch to fibrillar muscle fate in insects. Nature 2011; 479, 406–409. 10.1038/nature10559 22094701

[11] Bernstein SI, O'Donnell PT, Cripps RM. Molecular genetic analysis of muscle development, structure and function in Drosophila. Int. Rev. Cytol. 1993; 43: 63–152. 10.1016/s0074-7696(08)61874-4 8449665

[12] Bryantsev AL, Duong S, Brunetti TM, Chechenova MB, Lovato TL, Nelson C et al Extradenticle and Homothorax control adult muscle fiber identity in Drosophila. Developmental Cell. 2012; 23: 664–673. 10.1016/j.devcel.2012.08.004 22975331 PMC3575643

[13] Bryantsev AL, Baker PW, Lovato TL, Jaramillo MS, Cripps RM. Differential requirements for Myocyte enhancer factor-2 during adult myogenesis in Drosophila. Developmental Biology 2012; 361: 191–207. 10.1016/j.ydbio.2011.09.031 22008792 PMC3246536

[14] Soler C, Han J, Taylor MV. The conserved transcription factor Mef2 has multiple roles in adult Drosophila musculature formation. Development 2012; 139: 1270–1275. 10.1242/dev.077875 22357930

[15] Mas J-A, Garcia-Zaragoza E, Cervera M. Two functionally identical modular enhancers in Drosophila troponin T gene establish the correct protein levels in different muscle types. Mol Biol Cell 2004; 15: 1931–1945. 14718560 10.1091/mbc.E03-10-0729 PMC379288

[16] Marin M-C, Rodriguez J-R, Ferrus A. Transcription of Drosophila Troponin I gene is regulated by two conserved, functionally identical, synergistic elements. Mol Biol Cell 2004; 15: 1185–1196. 14718563 10.1091/mbc.E03-09-0663 PMC363105

[17] Herranz R, Diaz-Castillo C, Nguyen TP, Lovato TL, Cripps RM, Marco R. Characterization of the whole Troponin C gene repertoire in *Drosophila melanogaster* . Gene Exp. Patt. 2004; 4: 183–190. 10.1016/j.modgep.2003.09.008 15161098

[18] Qiu F, Lakey A, Agianian B, Hutchings A, Butcher GW, Labeit S, et al Troponin C in different insect muscle types: identification of an isoform in Lethocerus, Drosophila and Anopheles that is specific to asynchronous flight muscle in the adult insect. Biochem. J. 2003; 371: 811–821. 12558500 10.1042/BJ20021814 PMC1223341

[19] Huang AM, Rehm EJ, Rubin GM. Quick preparation of genomic DNA from Drosophila. Cold Spring Harb Protoc. 2009; pdb.prot5198. 10.1101/pdb.prot5198 20147141

[20] Thummel CS, Pirrotta V. New pCaSpeR P element vectors. Drosoph. Inf. Serv. 1992; 71: 150.

[21] Horton RM. PCR-mediated recombination and mutagenesis. Mol Biotechnol 1995; 3: 93–99. 7620981 10.1007/BF02789105

[22] Kelly Tanaka KK, Bryantsev AL and Cripps RM. Myocyte enhancer factor-2 and Chorion factor-2 collaborate in activation of the myogenic program in Drosophila. Mol. Cell. Biol. 2008; 28: 1616–1629. 18160709 10.1128/MCB.01169-07 PMC2258795

[23] Rubin GM, Spradling AC. Genetic transformation of Drosophila with transposable element vectors. Science 1982; 218: 348–353. 6289436 10.1126/science.6289436

[24] Bischof J, Maeda RK, Hediger M, Karch F, Basler K. An optimized transgenesis system for Drosophila using germ-line-specific phiC31 integrases. Proc. Natl. Acad. Sci. USA. 2007; 104: 3312–3317. 17360644 10.1073/pnas.0611511104 PMC1805588

[25] Cripps RM, Black BL, Zhao B, Lien C-L, Schulz RA, Olson EN The myogenic regulatory gene *Mef2* is a direct target for transcriptional activation by Twist during Drosophila myogenesis. Genes Dev. 1998; 12: 422–434. 9450935 10.1101/gad.12.3.422 PMC316486

[26] Cripps RM, Lovato TL, Olson EN. Positive autoregulation of the *Myocyte enhancer factor-2* myogenic control gene during somatic muscle development in Drosophila. Dev. Biol. 2004; 267: 536–547. 15013812 10.1016/j.ydbio.2003.12.004

[27] Gossett LA, Kelvin DJ, Sternberg EA, Olson EN. A new myocyte-specific enhancer-binding factor that recognizes a conserved element associated with multiple muscle-specific genes. Mol Cell Biol 1989; 9: 5022–5033. 2601707 10.1128/mcb.9.11.5022-5033.1989 PMC363654

[28] Kawasaki H, Hirose S, Ueda H. A simple and quick method to isolate nuclear extracts from pupae of *Drosophila melanogaster* . Cytotechnology 2005; 49: 67–70. 10.1007/s10616-005-5414-3 19003064 PMC3449746

[29] Belozerov VE, Lin ZY, Gingras AC, McDermott JC, Siu KWM. High-resolution protein interaction map of the *Drosophila melanogaster* p38 mitogen-activated protein kinase reveals limited functional redundancy. Mol Cell Biol 2012; 32: 3695–3706. 10.1128/MCB.00232-12 22801366 PMC3430203

[30] Morriss GR, Bryantsev AL, Chechenova M, LaBeau EM, Lovato TL, Ryan KM, Cripps RM. Analysis of skeletal muscle development in Drosophila In: Myogenesis: methods and protocols, DiMario J, ed. Springer, New York 2011. 10.1007/978-1-61779-343-1_8 PMC7480019 22130835

[31] Andres V, Cervera M, Mahdavi V. Determination of the consensus binding site for MEF2 expressed in muscle and brain reveals tissue-specific sequence constraints. J Biol Chem 1995; 270: 23246–2329. 7559475 10.1074/jbc.270.40.23246

[32] Kelly KK, Meadows SM, Cripps RM. Drosophila *Mef2* is an essential regulator of *Actin57B* transcription in cardiac, skeletal and visceral muscle lineages. Mech. Dev. 2002; 110: 39–50. 11744367 10.1016/s0925-4773(01)00586-x

[33] Sandmann T, Jensen LJ, Jakobsen JS, Karzynski MM, Eichenlaub MP, Bork P et al A temporal map of transcription factor activity: Mef2 directly regulates target genes at all stages of muscle development. Dev Cell 2006; 10: 797–807. 16740481 10.1016/j.devcel.2006.04.009

[34] Zinzen RP, Girardot C, Gagneur J, Braun, Furlong EEF. Combinatorial binding predits spatio-temporal cis-regulatory activity. Nature 2009; 462: 65–70. 10.1038/nature08531 19890324

[35] Baker PW, Kelly Tanaka KK, Klitgord N, Cripps RM. Adult myogenesis in *Drosophila melanogaster* can proceed independently of Myocyte enhancer factor-2. Genetics 2005; 170: 1747–1759. 15956678 10.1534/genetics.105.041749 PMC1449755

[36] Arredondo JJ, Ferreres RM, Maroto M, Cripps RM, Marco R, Bernstein SI et al Control of Drosophila paramyosin/miniparamyosin gene expression. J Biol Chem 2001; 276: 8278–8287. 11110792 10.1074/jbc.M009302200

[37] Lin M-H, Nguyen HT, Dybala C, Storti RV. Myocyte-specific enhancer factor 2 acts cooperatively with a muscle activator region to regulate Drosophila tropomyosin gene muscle expression. Proc Natl Acad Sci USA 1996; 93: 4623–4628. 8643453 10.1073/pnas.93.10.4623 PMC39328

[38] Brunetti TM, Fremin B, Cripps RM. Identification of *singles bar* as a direct transcriptional target of Drosophila Myocyte enhancer factor-2 and a regulator of adult myoblast fusion. Developmental Biology 2015; 401: 299–309. 10.1016/j.ydbio.2015.02.026 25797154 PMC4424145

[39] Morin S, Charron F, Robitaille L, Nemer M. GATA-dependent recruitment of MEF2 proteins to target promoters. EMBO Journal 2000; 19: 2046–2055. 10790371 10.1093/emboj/19.9.2046 PMC305697

[40] Mittler G, Butter F, Mann M. A SILAC-based DNA protein interaction screen that identified candidate binding proteins to functional DNA elements. Genome Res 2009; 19: 284–293. 10.1101/gr.081711.108 19015324 PMC2652210

[41] Cuomo A, Sanfilippo R, Vaccari T, Bonaldi T Proteomics meets genetics: SILAC labeling of *Drosophila melanogaster* larvae and cells for in vivo functional studies. Methods Mol Biol 2014; 1188: 293–311. 10.1007/978-1-4939-1142-4_21 25059620

[42] Lania L, Majello B and DeLuca P. Transcriptional regulation by the Sp family proteins. Int J Biochem Cell Biol 1997; 29: 731–742. 9570130 10.1016/s1357-2725(97)00094-0

[43] Barrio R, Shea MJ, Carulli J, Lipcow K, Gaul U, Frommer G et al The *spalt-related* gene of *Drosophila melanogaster* is a member of an ancient gene family, defined by the adjacent, region-specific homeotic gene *spalt* . Dev Genes Evol 1996; 206: 315–325. 10.1007/s004270050058 24173589

[44] Eldred CC, Katzemich A, Patel M, Bullard B, Swank DM. The roles of troponin C isoforms in the mechanical function of Drosophila indirect flight muscle. J Muscle Res Cell Motil 2014; 35: 211–223. 10.1007/s10974-014-9387-8 25134799 PMC4232998

